# Heart Rate Measurement Accuracy of Fitbit Charge 4 and Samsung Galaxy Watch Active2: Device Evaluation Study

**DOI:** 10.2196/33635

**Published:** 2022-03-01

**Authors:** Michael Nissen, Syrine Slim, Katharina Jäger, Madeleine Flaucher, Hanna Huebner, Nina Danzberger, Peter A Fasching, Matthias W Beckmann, Stefan Gradl, Bjoern M Eskofier

**Affiliations:** 1 Department Artificial Intelligence in Biomedical Engineering Friedrich-Alexander University Erlangen-Nürnberg Erlangen Germany; 2 Department of Gynecology and Obstetrics Erlangen University Hospital Erlangen Germany

**Keywords:** wearable validation, heart rate validation, Fitbit Charge 4, Samsung Galaxy Watch Active2, heart rate accuracy, fitness tracker accuracy, wearable accuracy, wearable, Fitbit, heart rate, fitness tracker, fitness, cardiovascular

## Abstract

**Background:**

Fitness trackers and smart watches are frequently used to collect data in longitudinal medical studies. They allow continuous recording in real-life settings, potentially revealing previously uncaptured variabilities of biophysiological parameters and diseases. Adequate device accuracy is a prerequisite for meaningful research.

**Objective:**

This study aims to assess the heart rate recording accuracy in two previously unvalidated devices: Fitbit Charge 4 and Samsung Galaxy Watch Active2.

**Methods:**

Participants performed a study protocol comprising 5 resting and sedentary, 2 low-intensity, and 3 high-intensity exercise phases, lasting an average of 19 minutes 27 seconds. Participants wore two wearables simultaneously during all activities: Fitbit Charge 4 and Samsung Galaxy Watch Active2. Reference heart rate data were recorded using a medically certified Holter electrocardiogram. The data of the reference and evaluated devices were synchronized and compared at 1-second intervals. The mean, mean absolute error, mean absolute percentage error, Lin concordance correlation coefficient, Pearson correlation coefficient, and Bland-Altman plots were analyzed.

**Results:**

A total of 23 healthy adults (mean age 24.2, SD 4.6 years) participated in our study. Overall, and across all activities, the Fitbit Charge 4 slightly underestimated the heart rate, whereas the Samsung Galaxy Watch Active2 overestimated it (−1.66 beats per minute [bpm]/3.84 bpm). The Fitbit Charge 4 achieved a lower mean absolute error during resting and sedentary activities (seated rest: 7.8 vs 9.4; typing: 8.1 vs 11.6; laying down [left]: 7.2 vs 9.4; laying down [back]: 6.0 vs 8.6; and walking slowly: 6.8 vs 7.7 bpm), whereas the Samsung Galaxy Watch Active2 performed better during and after low- and high-intensity activities (standing up: 12.3 vs 9.0; walking fast: 6.1 vs 5.8; stairs: 8.8 vs 6.9; squats: 15.7 vs 6.1; resting: 9.6 vs 5.6 bpm).

**Conclusions:**

Device accuracy varied with activity. Overall, both devices achieved a mean absolute percentage error of just <10%. Thus, they were considered to produce valid results based on the limits established by previous work in the field. Neither device reached sufficient accuracy during seated rest or keyboard typing. Thus, both devices may be eligible for use in respective studies; however, researchers should consider their individual study requirements.

## Introduction

### Background

Wearables such as smart watches and fitness trackers enable data recording in real-life settings, where biomedical signals cannot be easily captured with conventional or clinical devices. They can provide unobtrusive, economic, high-resolution, longitudinal recording capabilities for various signals, including accelerometer and photoplethysmogram (PPG) data [1,2]. This makes them particularly interesting for use in longitudinal medical studies and biomedical research. Observational studies especially benefit from the unobtrusive and longitudinal recording characteristics of fitness trackers and wearables across a variety of medical disciplines [3-9]. In addition to clinical studies, fitness trackers are also usable in other applications. These include activity feedback, activity promotion, weight management, disease monitoring, disease diagnostics, stress and sleep monitoring, and health care surveillance [10-15].

An important prerequisite for the use of wearables in studies and connected applications is adequate accuracy and, thus, data quality. Without sufficient validation, the reliability of the recorded data is unknown, which is the case for many modern end consumer devices. Thus, stringent upfront validation is not only a necessity for meaningful research but also prospective applications.

### Related Work

The Fitbit Charge HR series (Fitbit) is one of the most frequently validated devices. The study by Lee [16] reviewed the first device generation with 10 college students under free-living conditions. Each participant was asked to conduct normal day activities for 8 hours, and the heart rate (HR) was recorded and evaluated every minute against a Polar HR chest strap monitor. They concluded that the device was not accurate, with a mean absolute percentage error (MAPE) of 9.17% (SD 10.9%) when worn on the nondominant hand. Brazendale et al [17] evaluated the HR measurements of 39 children. The evaluation was performed on a per-minute basis, and the MAPE was reported as 6.9%. Thus, the authors concluded that wearable fitness trackers provide HR measurements comparable with a criterion field–based measure.

The data of 50 intensive care unit patients monitored over 24 hours were used for evaluation by Kroll et al [15]. They recorded HR values every 5 minutes and identified a median difference of 1 beats per minute (bpm) between the derived HR of the fitness tracker and the electrocardiogram (ECG)–derived HR.

A higher comparison frequency was chosen by Jo et al [18]. By measuring and comparing the HR every second, 24 participants completed a 77-minute protocol comprising several activities, including cycling, walking, jogging, running, and other sports exercises. A 12-lead ECG served as the criterion device. The authors reported a mean bias of −8.8 bpm and concluded that the device by Fitbit does not satisfy the validity criteria, particularly during higher exercise intensities.

The second release of the Fitbit Charge HR series was evaluated by Reddy et al [19], Thomson et al [20], and Benedetto et al [21], yielding different results on the device accuracy. To the best of our knowledge, the only validation study for Fitbit Charge 3 was performed by Muggeridge et al [22], who stated that the Fitbit Charge 3 performed well only during resting and walking-like conditions but otherwise assessed the accuracy to be overall poor.

To the best of our knowledge, no validation studies on the Samsung Galaxy Watch Active series exist as of today. However, other Samsung smart watches have been validated in the past. The measurements of the Samsung Gear S were investigated by Wallen et al [23], with 22 participants in rest, walking, running, and cycling. Out of a total of 4 devices, Samsung Gear S demonstrated the greatest variability in HR measurements. Shcherbina et al [24] examined the Samsung Gear S2 among 6 other devices with 60 participants from diverse backgrounds, performing a range of activities, including sitting, walking, running, and cycling. Of the validated devices in the study, Samsung Gear S2 showed the highest overall error, particularly during sitting. In another study by El-Amrawy and Nounou [25], the device also showed the lowest accuracy compared with 17 other devices and a clinical pulse oximeter as a criterion device.

### Objective

The validation study presented here was conducted as an initial groundwork for a large-scale observational study in obstetrics. As a pilot study, we aim to assess the performance of the selected devices in a healthy population to initially determine eligibility for longitudinal medical studies in general. We were particularly interested in the performance and accuracy of HR measurements, which are analyzed in detail in the following sections. This work is the first to validate the Fitbit Charge 4 and Samsung Galaxy Watch Active2 (Samsung Group).

## Methods

### Overview

Details on the participants, experimental procedure, used devices, validation metrics, processing, and evaluation are outlined in the following sections. Where applicable and possible, we adhered to several common grounds, guidelines, and best practices for wearable HR validation, which have been published in the more recent past [2,12,26].

### Ethics Approval

The study was approved by the ethics committee of Friedrich–Alexander Universität Erlangen-Nürnberg (106_13 B). The participants provided informed consent to participate.

### Recruitment

Recruitment was conducted via mailing lists and direct contact. We were unable to perform a power calculation for sample size estimation as the selected devices have not been investigated in the past, and thus, no information on effect sizes or variances was available. Consequently, we aimed at a sample size of approximately 20 to 25 participants, which is in line with previous HR evaluation studies [18,19,22,27,28]. Exclusion criteria was a major underlying medical condition affecting the participants’ physical capability or increasing the risk of injury. Assessment was conducted using the Physical Activity Readiness Questionnaire [29]. Ultimately, 23 participants were recruited.

### Devices and Gold Standard

We aimed to investigate the accuracy of Fitbit Charge 4 and Samsung Galaxy Watch Active2. Fitbit Charge 4 was released in March 2020. According to the manufacturer, it has a battery runtime of up to 7 days, which makes it particularly interesting for longitudinal studies [30].

The Galaxy Watch Active2 is a smart watch using Tizen OS as the operating system. Its PPG sensor uses 8 photodiodes [31]. The smart watch is available in several sizes and editions; our study used a 40 mm–sized version without long-term evolution. Furthermore, the device is able to record the ECGs. It is possible to derive blood pressure measurements through the PPG sensor; an upfront validation with a blood pressure cuff is required beforehand, and it is recommended to repeat this validation every 4 weeks [32,33]. Tizen OS is extendable, and a documentation of several available application programming interfaces (APIs) to create custom applications and interact with the device is available on the web [34]. This includes functions for accessing nearly all the built-in sensors. The available functions are not limited to the reading of HR and RR intervals but also allow raw data access to nearly all built-in sensors, particularly the PPG sensor. These features make the device interesting for medical studies, as their own algorithms for data processing can be used.

A Mind Media NeXus-10 MKI (Mind Media BV) was used as the gold standard. The ECG Holter device is a certified medical device of class 2a (EU). Data are transferred in real time via Bluetooth to a computer running a manufacturer-supplied software called Biotrace+ (Mind Media) [35], which displays and allows the export of HR, heart rate variability, and ECG data.

### Study Procedure

The study was conducted in an indoor laboratory environment on 7 different days between July 30, 2020, and September 21, 2020. As the data recording was conducted during summer without air conditioning, ambient temperatures were comparably high for Northern Bavaria, causing sweaty skin surfaces in some cases. This can induce additional noise, electrode loss, or affect the PPG signal measurement of wearable devices.

After receiving information on the study procedure and aims, participants filled out the activity readiness questionnaire and respective consent forms. Participants were then supplied with the 2 wearable devices and were asked to place 1 device on each arm, ensuring that the sensor was in good contact with the skin and that the devices were fitted comfortably on the arms. The study adviser determined which device should be placed onto which arm, and devices were equally placed on the left or right arm across all participants. As the Fitbit app has a setting to determine whether the device is placed on the dominant or nondominant arm, this setting was configured accordingly by the study adviser based on the participant information. No such setting exists for the Galaxy Watch Active2. Subsequently, the Mind Media NeXus-10 MKI’s electrodes were placed in a lead two position. To reduce noise, electrodes were placed on the torso, not the extremities. As the Holter device was equipped with a handbag-like body strap, it was hung over the shoulder of participants to increase freedom of movement. This positioning method was supported by the manufacturer. Recording started at least 30 seconds after placement of the electrodes, ensuring sufficient time for adaption for both wearables and the ECG algorithms.

The participants conducted an experimental protocol covering 10 subsequent tasks. Each task lasted between 1 and 2 minutes. The protocol anticipated a total length of 15 minutes. The chosen activities originate from activity recommendations for women with pregnancies, who are the prospective target group in our anticipated larger study. Participants were asked to conduct activities at their own pace to resemble activities as they would be conducted *free living* by the target group. With transitions between the individual study protocol phases, the recordings had an average duration of 19:03 minutes. We tried to minimize transition or relaxation phases between activities to ensure that the respective HR levels were similar between adjunct activities. If minor slack times (usually <10 seconds) occurred between activities (eg, because of instructions by the study adviser or a move of position between activities), these slack times were not included in the individual activity analysis. Our overall goal was to initially start with resting and sedentary activities (seated rest, typing, laying down [left], and laying down [back]), then continually increase HR using low intensity (standing up and walking at a slow pace) and high intensity (walking at brisk pace, climbing stairs, and squat work out) activities. The full list of activities and tasks is presented in Table 1.

**Table 1:** List of conducted activities.

Activities	Duration (minute)	Description
Seated rest	2	Sit comfortably on a chair while breathing normally, without any physical movements Considered resting or baseline condition
Keyboard typing	1.5	Type a neutral text on a computer keyboard provided by the study advisor Aims to assess the effects of hand movement without general body movement
Laying on left side	1.5	Lay on the left body side on a flat mattress
Laying on back	1.5	Turn and lay down fully on the back
Standing up	1	Stand up and maintain an upright position without movement
Walking at a slow pace	1	Walk slowly and naturally around the laboratory at one’s own pace
Walking at a brisk pace	2	Increase the walking speed to the maximum walking speed without running at one’s own pace
Climbing stairs	1.5	Climb stairs up and down at one’s own pace Simulation of a workout for a woman with pregnancy
Squat workout	1.5	Conduct squats at one’s own pace Simulation of a workout for a woman with pregnancy
Seated rest	1.5	Sit down directly after the workout, relax your breathing, and remain without motion. Aims to assess drastic changes in heart rate from high activity to rest

### Data Recording and Processing

#### Fitbit Charge 4

To increase the sampling frequency of the Fitbit Charge 4, the device was set to the training mode before the first study run. This produced a HR measurement every 1 to 5 seconds, thus resulting in a sampling frequency between 0.2 and 1 Hz. As the fitness tracker is linked to a user account in Fitbit’s cloud, the data were accessed through the Fitbit Web API using representational state transfer queries and the Postman software. The API only provides access to HR measurements, and no PPG raw data or RR intervals are provided.

To compare data on a per-second basis, the HR values required upsampling. When not provided with a HR measurement every second, missing values were imputed using the next available HR value.

#### Samsung Galaxy Watch Active2

A custom application for Samsung’s Tizen OS was developed. The *Human Activity Monitor API* was used to retrieve HR and RR intervals. All retrieved data were saved in JSON format to files and downloaded to a computer. The *Human Activity Monitor API* provides HR and RR interval data with a sampling rate of 25 Hz (the provided callback function to the *humanactivitymonitor.start* function is called every 40 milliseconds). However, the data are inconclusive: the HR changes more frequently than physiologically explainable; that is, the API provides up to 5 HR changes (from 86 to 85 to 86 to 85 to 84) within a time frame as small as 300 milliseconds. At the same time, the reported RR interval occasionally remains unchanged over periods >10 seconds. Thus, we decided to sample the HR and RR interval data at 1 Hz. A minority of the data was sampled at a higher frequency and manually downsampled to 1 Hz.

#### Mind Media NeXus-10 MKI

The criterion device recorded ECG data at a sampling frequency of 256 Hz. Furthermore, it contained internal peak detection algorithms, also providing derived HR and RR interval data at 32 Hz. As stated before, data were transferred via Bluetooth from the Holter device to a computer running Mind Media Biotrace+. The data were then exported from the Mind Media Biotrace+ software as a CSV file. Activity sections were recorded and annotated by the study adviser during the study execution in software running on a laptop computer.

Owing to the nature of the study protocol, some activities were prone to noise. Particularly during squats and stair climbing, ECGs were sometimes noisy, and the manufacturer-supplied software was apparently unable to correctly identify R peaks, resulting in erratic and evidently wrong HR and RR interval data. This was particularly true for squat and stair-climbing activities.

To cope with this issue, the raw criterion ECG was again processed in Python, using the ECG function from the BioSPPY library [36]. Subsequently, the R peaks were manually revised by a human annotator and corrected. We then used a self-developed function to extract the HR from the RR intervals.

Finally, the data were downsampled to 1 Hz. If >1 HR value occurred during 1 second (as the HR was >60 bpm), the respective values were averaged.

#### Data Exclusion

Although manual data processing was applied to ensure high data quality, some recorded criterion ECGs were too noisy and unusable for comparison. Data were excluded if adequate criterion device recordings were unavailable but not if the measured data of the examined devices were evidently incorrect, as this situation could also appear in real-life use.

As a result, data of 3 participants (IDs 6, 8, and 23) had to be completely excluded. In addition, data of 2 participants were excluded for the *squat* and *walking stairs* activity (ID 5 and ID 7). After the *squat* activity, electrodes of 2 participants (ID 2 and ID 7) detached, and thus, no data were available. As stated before, a detailed overview of the conducted manual data correction and excluded activities of individual participants is provided in Table 2.

**Table 2:** Data exclusion and annotation.

Participant	Seated rest	Keyboard typing	Laying on left side	Laying on back	Standing up	Walking slow	Walking brisk	Stairs	Squats	Seated rest
1	Original^a^	Original	Annotated^b^	Original	Annotated	Original	Original	Original	Original	Original
2	Original	Original	Original	Original	Original	Original	Original	Original	Original	Excluded^c^
3	Original	Original	Annotated	Annotated	Annotated	Original	Annotated	Annotated	Original	Original
4	Original	Annotated	Annotated	Annotated	Original	Annotated	Annotated	Annotated	Annotated	Original
5	Original	Original	Annotated	Original	Original	Annotated	Annotated	Excluded	Excluded	Original
6	Excluded	Excluded	Excluded	Excluded	Excluded	Excluded	Excluded	Excluded	Excluded	Excluded
7	Original	Original	Original	Original	Original	Annotated	Annotated	Excluded	Excluded	Excluded
8	Excluded	Excluded	Excluded	Excluded	Excluded	Excluded	Excluded	Excluded	Excluded	Excluded
9	Original	Original	Original	Original	Original	Original	Original	Original	Original	Original
10	Original	Original	Original	Original	Annotated	Original	Original	Annotated	Original	Original
11	Original	Original	Original	Annotated	Original	Original	Original	Original	Annotated	Original
12	Original	Original	Original	Original	Original	Original	Original	Original	Original	Original
13	Original	Original	Original	Original	Original	Original	Original	Original	Original	Original
14	Original	Original	Original	Original	Original	Original	Original	Original	Annotated	Original
15	Original	Original	Original	Original	Original	Original	Original	Original	Annotated	Original
16	Original	Original	Original	Original	Annotated	Original	Original	Original	Original	Original
17	Original	Original	Original	Original	Original	Original	Original	Original	Original	Original
18	Annotated	Original	Original	Original	Annotated	Original	Original	Original	Original	Original
19	Original	Original	Original	Original	Original	Original	Original	Original	Annotated	Original
20	Original	Original	Original	Original	Annotated	Original	Original	Original	Annotated	Original
21	Original	Original	Original	Original	Original	Original	Original	Original	Original	Original
22	Original	Original	Original	Original	Original	Original	Original	Original	Original	Original
23	Excluded	Excluded	Excluded	Excluded	Excluded	Excluded	Excluded	Excluded	Excluded	Excluded

^a^Represents original data.

^b^Represents manually annotated data.

^c^Represents excluded activity participant combinations.

#### Data Synchronization

As the software of the validated fitness trackers is mostly closed source, their exact time measurement and determination mechanism are unknown. Furthermore, the on-device signal processing may cause additional delays. Therefore, we did not rely on exact time stamps for device synchronization but instead used another synchronization technique.

Synchronization of the signals was performed on the previously downsampled signals of all 3 devices (1 Hz, ie, 1 HR value per second). We conducted the synchronization between the individual validated devices and our HR reference by maximizing the Pearson correlation coefficient (PCC). The measurements were then shifted by the respectively determined time delays. This provided very similar and, in many cases, equal results to a shift through cross-correlation but showed better visual and metric results in a minority of edge cases.

### Statistical Analysis

All statistical analyses were conducted in Python (version 3.8.7) on a Windows 10 machine using Numpy 1.19.5 [37], Scipy 1.6.0 [38], Pandas 1.2.0 [39], and Pingouin 0.3.11 [40]. Raw data and respective scripts are available from the authors upon request.

Absolute error analysis was conducted using the mean absolute error (MAE) and MAPE as key metrics. We defined MAE as the average absolute distance between the HR of the validated device and the criterion device. MAPE is the percentage difference between the reference and the respective device values. The limits of agreement and mean error (bias) were derived from Bland–Altman plots, which also visually aided in the interpretation of the results. Correlation analysis was performed using the Lin concordance correlation coefficient (CCC), as suggested by Sartor et al [12,41,42]. PCC was additionally reported for completeness but not analyzed.

## Results

### Participants

In total, 23 healthy individuals participated in the study (n=10, 43% women and n=13, 57% men). The demographics and details of the participants are shown in Table 3. Most participants were university students and staff members. Given the location of the university, Fitzpatrick skin type 2 was overrepresented (3×type 1, 15×type 2, 2×type 3, 1×type 4, and 2×type 5).

**Table 3:** Demographics and details of the participants.

Characteristics	Values, minimum	Values, maximum	Values, mean (SD)
Age (years)^a^	20	36	24.2 (4.6)
Height (cm)	156	193	175.7 (10.48)
Body weight (kg)^b^	50	88	71 (12.75)

^a^One participant did not provide his or her date of birth.

^b^One participant did not provide his or her body weight.

### HR Measurement

The key results of this validation study are summarized in Table 4. In total and across the entire experiment duration (ie, all activities), both devices achieved very similar values for MAE, MAPE, and PCC. Although the Fitbit Charge 4 slightly underestimated the HR by −1.66 bpm (bias), the Samsung Galaxy Watch Active2 overestimated the HR by 3.84 bpm (bias).

In resting and sedentary activities (seated rest, typing, and laying down) and slow walking, the Fitbit Charge 4 achieved lower absolute and absolute percentage error rates. During standing up and all other physical activities, the Samsung Galaxy Watch Active2 outperformed the Fitbit Charge 4.

A particularly high bias (ie, mean difference) was observed by the Fitbit Charge 4 during standing up (−7.95 bpm) and squats (−12.52 bpm). The Samsung Galaxy Watch Active2’s highest bias was measured during typing (8.63 bpm) and laying down on the left side (6.01 bpm).

**Figure 1:**
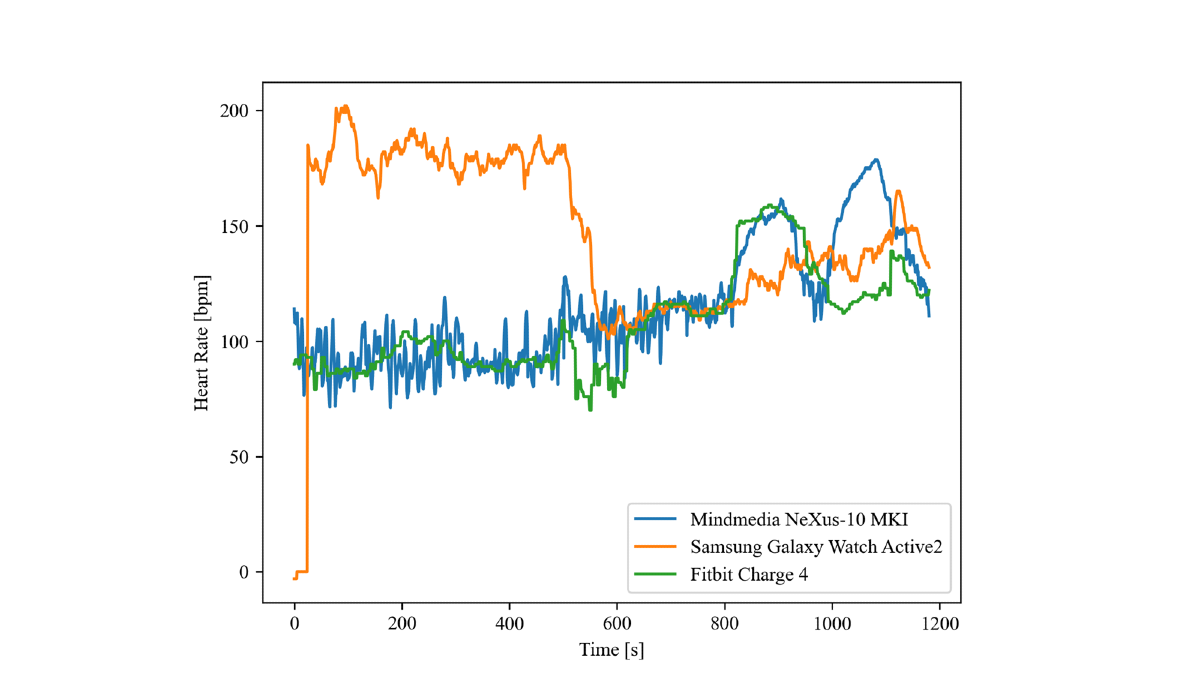
Heart rate measurement of participant 20. Samsung Galaxy Watch Active2 recorded no or excessive heart rate values during the first 5 activities.

The level of agreement of the Samsung Galaxy Watch Active2 is particularly broad during activities 1 to 5. The cause was a non- or excessive recorded HR in participant 20 during these activities, where the device recorded an average HR of 146, 148, 181, and 176 bpm. This HR trend is displayed in Figure 1. If this participant was excluded from the data analysis, the metrics drastically improved: MAE and MAPE were consistently lower than those of the Fitbit device for activities 1 to 5, and both widths of limits of agreement and bias were reduced significantly.

CCC was consistently higher in the Samsung Galaxy Watch Active2. Both devices achieved particularly low scores (<0.250) during typing and slow walking. The resting phase resulted in the highest individual activity of CCC in both devices.

**Figure 2:**
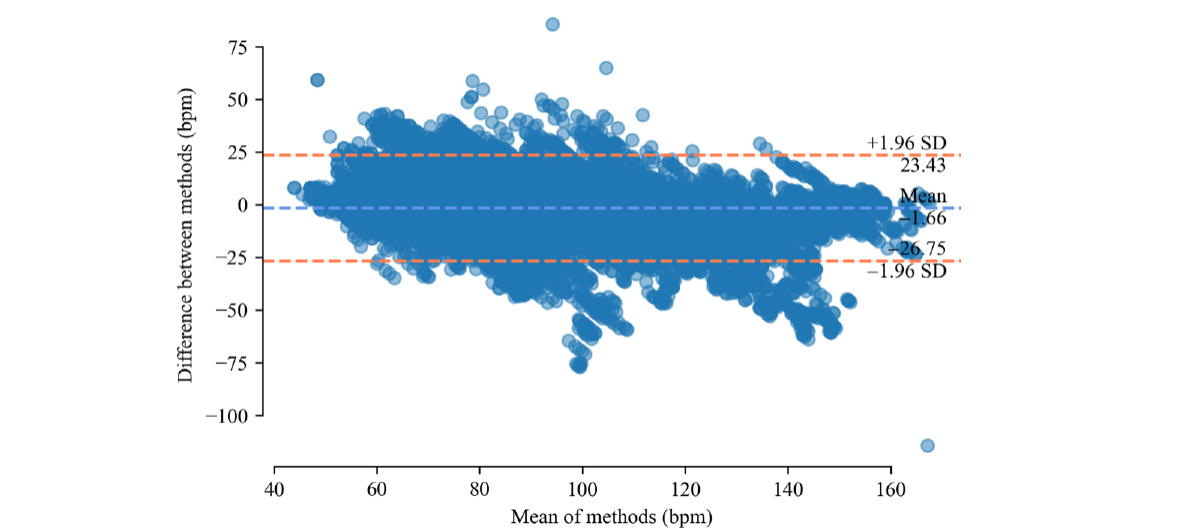
Bland-Altman plot for heart rate difference between Mind Media NeXus-10 MKI and Fitbit Charge 4 across all participants and activities.

**Figure 3:**
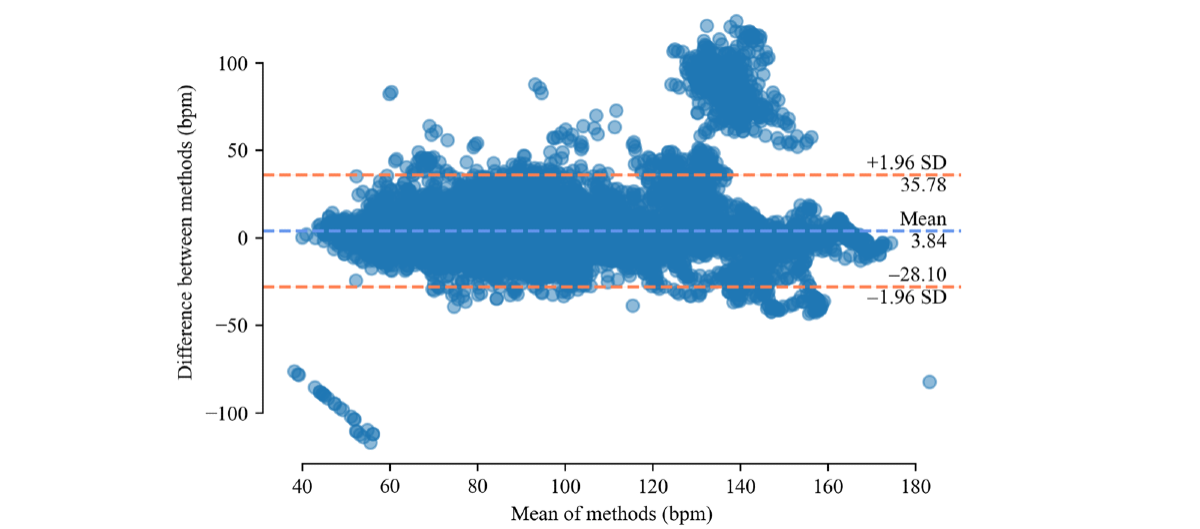
Bland-Altman plot for heart rate difference between Mind Media NeXus-10 MKI and Samsung Galaxy Watch Active2 across all participants and activities.

Bland–Altman plots for both devices are shown in Figures 2 and 3. A large cluster of points in the top-right section of Figure 3 is particularly noticeable. These data points are a result of the previously mentioned mismeasurement of the Samsung Galaxy Watch Active2. If participant 20 is excluded from the data set, the respective cluster disappears from the plot.

**Table 4:** Validation metrics of heart rate measurements across different activities and devices.

Activity (metrics)			Mind Media NeXus-10 MKI		Fitbit Charge 4		Samsung Galaxy Watch Active2
**Overall (0)**							
	Values (bpm^a^), mean (SD)	92.48 (22.34)		90.85 (18.75)		96.26 (22.55)	
	MAE^b^ (bpm)	N/A^c^		8.589		8.13	
	MAPE^d^	N/A		9.74		9.419	
	CCC^e^	N/A		0.805		0.847	
	PCC^f^	N/A		0.839		0.85	
	Bias (bpm)	N/A		−1.66		3.84	
	LoA^g^ (bpm)	N/A		−26.75 to 23.43		−28.1 to 35.78	
**Seated rest (1)**							
	Values (bpm), mean (SD)	75.65 (7.17)		78.75 (3.88)		79.89 (9.43)	
	MAE (bpm)	N/A		7.829		9.381	
	MAPE	N/A		11.801		12.013	
	CCC	N/A		0.203		0.508	
	PCC	N/A		0.257		0.556	
	Bias (bpm)	N/A		3.36		4.41	
	LoA (bpm)	N/A		−18.98 to 25.70		−40.5 to 49.33	
**Typing (2)**							
	Values (bpm), mean (SD)	78.53 (6.23)		79.44 (3.1)		87.32 (5.84)	
	MAE (bpm)	N/A		8.139		11.62	
	MAPE	N/A		11.24		14.815	
	CCC	N/A		0.057		0.207	
	PCC	N/A		0.094		0.211	
	Bias (bpm)	N/A		0.79		8.63	
	LoA (bpm)	N/A		−1.96 to 22.14		−33.18 to 50.43	
**Laying down (left; 3)**							
	Values (bpm), mean (SD)	73.57 (9.17)		75.25 (4.48)		79.74 (8.94)	
	MAE (bpm)	N/A		7.245		9.35	
	MAPE	N/A		9.938		11.901	
	CCC	N/A		0.382		0.624	
	PCC	N/A		0.507		0.663	
	Bias (bpm)	N/A		2.13		6.01	
	LoA (bpm)	N/A		−17.49 to 21.75		−31.6 to 43.62	
**Laying down (back; 4)**							
	Values (bpm), mean (SD)	68.49 (7.75)		68.51 (3.61)		73.52 (5.25)	
	MAE (bpm)	N/A		6.034		8.622	
	MAPE	N/A		9.062		11.242	
	CCC	N/A		0.249		0.554	
	PCC	N/A		0.358		0.609	
	Bias (bpm)	N/A		0.03		4.82	
	LoA (bpm)	N/A		−16.62 to 16.67		−33.7 to 43.34	
**Standing up (5)**							
	Values (bpm), mean (SD)	88.83 (11.76)		81.03 (6.69)		88.68 (9.14)	
	MAE (bpm)	N/A		12.25		8.976	
	MAPE	N/A		13.302		9.988	
	CCC	N/A		0.253		0.519	
	PCC	N/A		0.345		0.62	
	Bias (bpm)	N/A		−7.95		0.52	
	LoA (bpm)	N/A		−37.83 to 21.93		−30.19 to 31.23	
**Walking slow (6)**							
	Values (bpm), mean (SD)	86.24 (6.38)		87.3 (3.86)		90.99 (3.53)	
	MAE (bpm)	N/A		6.781		7.742	
	MAPE	N/A		8.118		9.046	
	CCC	N/A		0.15		0.18	
	PCC	N/A		0.188		0.24	
	Bias (bpm)	N/A		1.2		4.94	
	LoA (bpm)	N/A		17.77 to 20.16		−15.45 to 25.34	
**Walking fast (7)**							
	Values (bpm), mean (SD)	100.22 (6.64)		99.11 (4.42)		102.82 (4.92)	
	MAE (bpm)	N/A		6.094		5.829	
	MAPE	N/A		6.364		6.292	
	CCC	N/A		0.348		0.439	
	PCC	N/A		0.408		0.516	
	Bias (bpm)	N/A		−0.98		2.86	
	LoA (bpm)	N/A		−20.68 to 18.73		−16.8 to 22.51	
**Stairs (8)**							
	Values (bpm), mean (SD)	119.67 (13.83)		115.54 (9.45)		121.14 (10.19)	
	MAE (bpm)	N/A		8.811		6.879	
	MAPE	N/A		7.605		6.157	
	CCC	N/A		0.634		0.691	
	PCC	N/A		0.803		0.812	
	Bias (bpm)	N/A		−3.99		1.28	
	LoA (bpm)	N/A		−25.61 to 17.63		−19.14 to 21.7	
**Squats (9)**							
	Values (bpm), mean (SD)	129.05 (11.87)		116.6 (7.72)		130.26 (7.28)	
	MAE (bpm)	N/A		15.737		6.163	
	MAPE	N/A		11.976		5.51	
	CCC	N/A		0.29		0.61	
	PCC	N/A		0.335		0.668	
	Bias (bpm)	N/A		−12.52		1.18	
	LoA (bpm)	N/A		−50.46 to 25.42		−20.55 to 22.92	
**Resting (10)**							
	Values (bpm), mean (SD)	106.17 (16.79)		102.8 (11.25)		105.82 (14.12)	
	MAE (bpm)	N/A		9.612		5.618	
	MAPE	N/A		9.749		5.82	
	CCC	N/A		0.65		0.845	
	PCC	N/A		0.752		0.872	
	Bias (bpm)	N/A		−3.45		−0.38	
	LoA (bpm)	N/A		−28.52 to 21.61		−15.75 to 15.00	

^a^bpm: beats per minute.

^b^MAE: mean absolute error.

^c^N/A: not applicable.

^d^MAPE: mean absolute percentage error.

^e^CCC: Lin concordance correlation coefficient.

^f^PCC: Pearson correlation coefficient.

^g^LoA: limits of agreement.

## Discussion

### Comparison With Previous Work

Our study aimed to evaluate 2 consumer wearable devices in healthy participants over a range of activities. The results from our study indicate that both devices achieved a MAPE <10%. Although no previous work exists for the Samsung Galaxy Watch Active2, our results are somewhat in line with previous validation trials for the Fitbit Charge series.

A previous evaluation of the Fitbit Charge 3 by Muggeridge et al [22] used a notably different experimental protocol, emphasizing strenuous activities (with a focus on treadmill running, sprinting, and cycling). The authors report an overall MAPE of 7.37 (as compared with 9.74 in our study) and note that the device underestimates the HR by −7 bpm (here, −1.66 bpm). Overall, the study states that the Fitbit device performs poorly during high-intensity activities and results in a higher error in that area. In our study, the Fitbit device’s mean bias was highest while climbing stairs and squatting, with a bias of −3.99 bpm and −12.52 bpm, respectively.

Reviewing studies on the Fitbit Charge 2, underestimations of the HR have been reported by several other studies [19,21,43]. The study by Baek et al [44] only reported this underestimation in the <100 bpm category and an overestimation of >120 bpm. With respect to the MAPE, the study by Reddy et al [19] reported a value of 11.33%, and the study by Nelson et al [43] reported a value of 5.96% across all activities. Our measured CCC of 0.805 across all activities is lower than the CCC of 0.906 reported by Nelson et al [43] in a 24-hour period.

### Measurement Validity

Different validation definitions exist in the literature. Some prior studies have used an error rate of +5% to –5% as a limit, as it “approximates a widely accepted standard for statistical significance [...]” [24] and is “widely accepted” [19]. A limit of +10% to –10% is established by various organizations and institutions and has been equally used by other validation studies [43]. The latter value is also proposed by previously mentioned validation guidelines [12] and thus, used for further reference in our work.

Similarly, different interpretations of correlation coefficients have been used in the literature. Owing to the large number of different definitions, ranging from a *weak* or *poor* interpretation starting between <0.2, <0.50, and <0.9 [23,43,45], we refrain from the use of an exact definition.

In our study and across all activities, both devices achieved a MAPE <10% and, per definition, produced valid results. With respect to individual activities, neither device produced valid results for seated rest and typing activities. Furthermore, the Fitbit Charge 4 did not record valid data for the standing up and squat activities, and the Samsung Galaxy Watch Active2 produced invalid results for laying down in either of the 2 evaluated positions.

### Limitations

#### Participants and Demographic Structure

Our study mainly included healthy young participants aged between 20 and 36 years. Wearables may provide different validation results for older participants, particularly with respect to their skin properties and changes in the PPG curve. Furthermore, as most of our participants were local university students in middle Europe, Fitzpatrick skin types 1 to 3 were overrepresented in our study.

#### Selected Activities

The overall duration of individual activities was rather short, mostly because our aim was to set a low burden for study participation. Although the HR of all participants increased during the study duration (especially during the second half of the study), some participants may require a longer activity duration for optimal HR adaption. A shorter activity duration makes the collected data less meaningful and results in a lower number of recorded data points, thus decreasing statistical expressiveness.

As all activities were conducted consecutively and without breaks, splits between individual recorded activities always resulted in minor transitional phases. Some participants may react faster to the instructions of the study instructor than others. This leads to additional time slack between individual activities and may cause a slight metric profusion between the 2 subsequent activity metrics.

#### Laboratory Conditions and Environmental Factors

Although we aimed to replicate real-life activities as much as possible, our study was still conducted in a laboratory setting. Real-life use patterns may differ from those in our study and, as such, may have an impact on the accuracy of the investigated devices. Furthermore, our study was mostly conducted during warm summer days, and our laboratory was not equipped with air conditioning. Sweat is known to have an influence on ECG electrode conductance. It may also have an impact on PPG measurements by the examined wearable devices.

#### Data Annotation and Exclusion

Owing to various influencing factors—mainly ECG electrode loss, heat, selected activities, and other unknown skin factors—less data than anticipated were ultimately included in our study (20/23, 87% participants). A solid baseline (ground truth) was of the utmost importance in our study. Our manual data annotation of the criterion device data underlines this effort. As the annotation affects only the criterion device, it has no impact on the data recorded by the evaluated devices and, therefore, on future studies.

For participants 2, 5, and 7, only a subset of activities was included in our statistical analysis (Table 2). Although the respective individual activity metric averages reported in Table 4 do not include data for the respective activities, we did not exclude these individual participants for the overall metrics. This may lead to a minor bias toward resting and sedentary activities, as activities with higher physical activities were more prone to noise and, thus, data exclusion. Metrics only show minor changes if the data of these participants are excluded from the overall metric. The overall Fitbit Charge 4 MAE changed from 8.589 to 8.614 upon exclusion, and the Samsung Galaxy Watch Active2 MAE increased from 8.13 to 8.429.

The inclusion of data of participant 20 is controversial. A main argument for potential exclusion is that the data are clearly erroneous, and such data would be equally excluded in the study settings. On the other hand, faulty recordings may also occur in real-life settings. Excluding the data would lead to a positive bias in favor of the Samsung Galaxy Watch Active2 and, thus, to a nonobjective comparison. Therefore, we decided to include these data.

### Conclusions

We evaluated 2 previously unvalidated wearable devices by conducting a study featuring various activities and 23 participants. Throughout the entire experimental procedure, both devices achieved results just <10% MAPE and thus, presented acceptable HR measurement capabilities. The Fitbit Charge 4 outperformed the Samsung Galaxy Watch Active2 during resting and sedentary activities, and the Samsung device was more accurate during high-intensity activities. Neither device reached sufficient accuracy during seated rest and keyboard typing.

Our study was a prequel to a larger interdisciplinary study in obstetrics. Researchers should consider the intended use of wearable devices when reviewing validation studies and evaluating their respective findings with respect to their full requirements. This is not only the case for the experimental design but also for other aspects. Accuracy may not be the only decisive factor. Features such as raw data access, battery runtime, or additional sensors may be equally relevant for individual research.

## Abbreviations

API: application programming interface
bpm: beats per minute
CCC: Lin concordance correlation coefficient
ECG: electrocardiogram
HR: heart rate
MAE: mean absolute error
MAPE: mean absolute percentage error
PCC: Pearson correlation coefficient
PPG: photoplethysmography or photoplethysmogram

## References

[1] Zheng YL, Ding XR, Poon CC, Lo BP, Zhang H, Zhou XL, Yang GZ, Zhao N, Zhang YT (2014). Unobtrusive sensing and wearable devices for health informatics. IEEE Trans Biomed Eng.

[2] Nelson BW, Low CA, Jacobson N, Areán P, Torous J, Allen NB (2020). Guidelines for wrist-worn consumer wearable assessment of heart rate in biobehavioral research. NPJ Digit Med.

[3] Daskivich TJ, Houman J, Lopez M, Luu M, Fleshner P, Zaghiyan K, Cunneen S, Burch M, Walsh C, Paiement G, Kremen T, Soukiasian H, Spitzer A, Jackson T, Kim HL, Li A, Spiegel B (2019). Association of wearable activity monitors with assessment of daily ambulation and length of stay among patients undergoing major surgery. JAMA Netw Open.

[4] Lee JE, Lee DH, Oh TJ, Kim KM, Choi SH, Lim S, Park YJ, Park DJ, Jang HC, Moon JH (2018). Clinical feasibility of monitoring resting heart rate using a wearable activity tracker in patients with thyrotoxicosis: prospective longitudinal observational study. JMIR Mhealth Uhealth.

[5] Goodale BM, Shilaih M, Falco L, Dammeier F, Hamvas G, Leeners B (2019). Wearable sensors reveal menses-driven changes in physiology and enable prediction of the fertile window: observational study. J Med Internet Res.

[6] Shilaih M, Goodale BM, Falco L, Kübler F, De Clerck V, Leeners B (2018). Modern fertility awareness methods: wrist wearables capture the changes in temperature associated with the menstrual cycle. Biosci Rep.

[7] Shilaih M, de Clerck V, Falco L, Kübler F, Leeners B (2017). Pulse rate measurement during sleep using wearable sensors, and its correlation with the menstrual cycle phases, a prospective observational study. Sci Rep.

[8] Mishra T, Wang M, Metwally AA, Bogu GK, Brooks AW, Bahmani A, Alavi A, Celli A, Higgs E, Dagan-Rosenfeld O, Fay B, Kirkpatrick S, Kellogg R, Gibson M, Wang T, Hunting EM, Mamic P, Ganz AB, Rolnik B, Li X, Snyder MP (2020). Pre-symptomatic detection of COVID-19 from smartwatch data. Nat Biomed Eng.

[9] Quer G, Radin JM, Gadaleta M, Baca-Motes K, Ariniello L, Ramos E, Kheterpal V, Topol EJ, Steinhubl SR (2021). Wearable sensor data and self-reported symptoms for COVID-19 detection. Nat Med.

[10] Schembre SM, Liao Y, Robertson MC, Dunton GF, Kerr J, Haffey ME, Burnett T, Basen-Engquist K, Hicklen RS (2018). Just-in-time feedback in diet and physical activity interventions: systematic review and practical design framework. J Med Internet Res.

[11] Fawcett E, Van Velthoven MH, Meinert E (2020). Long-term weight management using wearable technology in overweight and obese adults: systematic review. JMIR Mhealth Uhealth.

[12] Sartor F, Papini G, Cox LG, Cleland J (2018). Methodological shortcomings of wrist-worn heart rate monitors validations. J Med Internet Res.

[13] Kristoffersson A, Lindén M (2020). Wearable sensors for monitoring and preventing noncommunicable diseases: a systematic review. Information.

[14] Van Hooren B, Goudsmit J, Restrepo J, Vos S (2020). Real-time feedback by wearables in running: current approaches, challenges and suggestions for improvements. J Sports Sci.

[15] Kroll RR, McKenzie ED, Boyd JG, Sheth P, Howes D, Wood M, Maslove DM, WEARable Information Technology for hospital INpatients (WEARIT-IN) study group (2017). Use of wearable devices for post-discharge monitoring of ICU patients: a feasibility study. J Intensive Care.

[16] Lee JM, An HS, Kang SK, Kim Y, Dinkel D (2016). Examining the validity of Fitbit charge HR for measuring heart rate in free-living conditions. Med Sci Sports Exerc.

[17] Brazendale K, Decker L, Hunt ET, Perry MW, Brazendale AB, Weaver RG, Beets MW (2019). Validity and wearability of consumer-based fitness trackers in free-living children. Int J Exerc Sci.

[18] Jo E, Lewis K, Directo D, Kim MJ, Dolezal BA (2016). Validation of biofeedback wearables for photoplethysmographic heart rate tracking. J Sports Sci Med.

[19] Reddy RK, Pooni R, Zaharieva DP, Senf B, El Youssef J, Dassau E, Doyle Iii FJ, Clements MA, Rickels MR, Patton SR, Castle JR, Riddell MC, Jacobs PG (2018). Accuracy of wrist-worn activity monitors during common daily physical activities and types of structured exercise: evaluation study. JMIR Mhealth Uhealth.

[20] Thomson EA, Nuss K, Comstock A, Reinwald S, Blake S, Pimentel RE, Tracy BL, Li K (2019). Heart rate measures from the Apple Watch, Fitbit Charge HR 2, and electrocardiogram across different exercise intensities. J Sports Sci.

[21] Benedetto S, Caldato C, Bazzan E, Greenwood DC, Pensabene V, Actis P (2018). Assessment of the Fitbit Charge 2 for monitoring heart rate. PLoS One.

[22] Muggeridge DJ, Hickson K, Davies AV, Giggins OM, Megson IL, Gorely T, Crabtree DR (2021). Measurement of heart rate using the polar OH1 and Fitbit charge 3 wearable devices in healthy adults during light, moderate, vigorous, and sprint-based exercise: validation study. JMIR Mhealth Uhealth.

[23] Wallen MP, Gomersall SR, Keating SE, Wisløff U, Coombes JS (2016). Accuracy of heart rate watches: implications for weight management. PLoS One.

[24] Shcherbina A, Mattsson CM, Waggott D, Salisbury H, Christle JW, Hastie T, Wheeler MT, Ashley EA (2017). Accuracy in wrist-worn, sensor-based measurements of heart rate and energy expenditure in a diverse cohort. J Pers Med.

[25] El-Amrawy F, Nounou MI (2015). Are currently available wearable devices for activity tracking and heart rate monitoring accurate, precise, and medically beneficial?. Healthc Inform Res.

[26] Düking P, Fuss FK, Holmberg HC, Sperlich B (2018). Recommendations for assessment of the reliability, sensitivity, and validity of data provided by wearable sensors designed for monitoring physical activity. JMIR Mhealth Uhealth.

[27] Parak J, Korhonen I (2014). Evaluation of wearable consumer heart rate monitors based on photopletysmography. Annu Int Conf IEEE Eng Med Biol Soc.

[28] Navalta JW, Montes J, Bodell NG, Salatto RW, Manning JW, DeBeliso M (2020). Concurrent heart rate validity of wearable technology devices during trail running. PLoS One.

[29] Thomas S, Reading J, Shephard RJ (1992). Revision of the physical activity readiness questionnaire (PAR-Q). Can J Sport Sci.

[30] Fitbit charge 4: advanced fitness tracker. Fitbit.

[31] Samsung India.

[32] Moon JH, Kang MK, Choi CE, Min J, Lee HY, Lim S (2020). Validation of a wearable cuff-less wristwatch-type blood pressure monitoring device. Sci Rep.

[33] Galaxy watch active 2. Samsung de.

[34] Get started with Tizen wearable applications. Tizen docs.

[35] Biofeedback, neurofeedback and qEEG. Mind Media - BioTrace+ Software.

[36] Welcome to BioSPPy - BioSPPy 0.6.1 documentation. BioSPPy.

[37] Harris CR, Millman KJ, van der Walt SJ, Gommers R, Virtanen P, Cournapeau D, Wieser E, Taylor J, Berg S, Smith NJ, Kern R, Picus M, Hoyer S, van Kerkwijk MH, Brett M, Haldane A, Del Río JF, Wiebe M, Peterson P, Gérard-Marchant P, Sheppard K, Reddy T, Weckesser W, Abbasi H, Gohlke C, Oliphant TE (2020). Array programming with NumPy. Nature.

[38] Virtanen P, Gommers R, Oliphant TE, Haberland M, Reddy T, Cournapeau D, Burovski E, Peterson P, Weckesser W, Bright J, van der Walt SJ, Brett M, Wilson J, Millman KJ, Mayorov N, Nelson AR, Jones E, Kern R, Larson E, Carey CJ, Polat İ, Feng Y, Moore EW, VanderPlas J, Laxalde D, Perktold J, Cimrman R, Henriksen I, Quintero EA, Harris CR, Archibald AM, Ribeiro AH, Pedregosa F, van Mulbregt P, SciPy 1.0 Contributors (2020). SciPy 1.0: fundamental algorithms for scientific computing in Python. Nat Methods.

[39] McKinney W (2010). Data structures for statistical computing in Python. 9th Python in Science Conference.

[40] Vallat R (2018). Pingouin: statistics in Python. J Open Source Softw.

[41] GitHub stylianos-kampakis/supervisedPCA-Python. https://github.com/stylianos-kampakis/supervisedPCA-Python.

[42] Lin LI (1989). A concordance correlation coefficient to evaluate reproducibility. Biometrics.

[43] Nelson BW, Allen NB (2019). Accuracy of consumer wearable heart rate measurement during an ecologically valid 24-hour period: intraindividual validation study. JMIR Mhealth Uhealth.

[44] Baek S, Ha Y, Park HW (2021). Accuracy of wearable devices for measuring heart rate during conventional and Nordic walking. PM R.

[45] Akoglu H (2018). User's guide to correlation coefficients. Turk J Emerg Med.

